# Are adolescents more vulnerable to the harmful effects of cannabis than adults? A placebo-controlled study in human males

**DOI:** 10.1038/tp.2016.225

**Published:** 2016-11-29

**Authors:** C Mokrysz, T P Freeman, S Korkki, K Griffiths, H V Curran

**Affiliations:** 1 Clinical Psychopharmacology Unit, Clinical Educational and Health Psychology, University College London, London, UK; 2 Behavioural and Clinical Neuroscience Institute, University of Cambridge, Cambridge, UK; 3 MRC Cognition and Brain Sciences Unit, University of Cambridge, Cambridge, UK

## Abstract

Preclinical research demonstrates that cannabinoids have differing effects in adolescent and adult animals. Whether these findings translate to humans has not yet been investigated. Here we believe we conducted the first study to compare the acute effects of cannabis in human adolescent (*n=*20; 16–17 years old) and adult (*n=*20; 24–28 years old) male cannabis users, in a placebo-controlled, double-blind cross-over design. After inhaling vaporized active or placebo cannabis, participants completed tasks assessing spatial working memory, episodic memory and response inhibition, alongside measures of blood pressure and heart rate, psychotomimetic symptoms and subjective drug effects (for example, ‘stoned', ‘want to have cannabis'). Results showed that on active cannabis, adolescents felt less stoned and reported fewer psychotomimetic symptoms than adults. Further, adults but not adolescents were more anxious and less alert during the active cannabis session (both pre- and post-drug administration). Following cannabis, cognitive impairment (reaction time on spatial working memory and prose recall following a delay) was greater in adults than adolescents. By contrast, cannabis impaired response inhibition accuracy in adolescents but not in adults. Moreover, following drug administration, the adolescents did not show satiety; instead they wanted more cannabis regardless of whether they had taken active or placebo cannabis, while the opposite was seen for adults. These contrasting profiles of adolescent resilience (blunted subjective, memory, physiological and psychotomimetic effects) and vulnerability (lack of satiety, impaired inhibitory processes) show some degree of translation from preclinical findings, and may contribute to escalated cannabis use by human adolescents.

## Introduction

An estimated 13% of 15–16-year olds in Europe and 23% of 15–17-year olds in the USA have taken cannabis in the previous year.^1, 2^ Globally the median age of first cannabis use falls between 18–19 years old,^3, 4^ indicating that approximately half of all cannabis users start before reaching adulthood.

The main psychoactive ingredient of cannabis, delta-9-tetrahydrocannabinol (THC), acts on the endocannabinoid (eCB) system, primarily as a partial agonist of the cannabinoid receptor CB1R. Studies with adult cannabis users have found altered eCB levels in cerebrospinal fluid^5^ and downregulated cortical CB1Rs,^6, 7^ relative to non-using controls. Although research into adolescent development of the eCB system remains in its infancy, it appears to undergo dynamic changes throughout adolescence,^8^ with evidence of increasing CB1R density continuing into late adolescence^9, 10^ (although also see Ellgren *et al.*^8^and Moore *et al.*^11^), and changing levels of eCBs in the prefrontal cortex and nucleus accumbens throughout adolescence.^8, 9^ The eCB system is also thought to have an important role in neural reorganization and maturational processes occurring during adolescence,^12, 13^ and has recently been implicated in the maturational pruning of glutamatergic synapses^14^ and development of GABA-ergic systems^15^ in the prefrontal cortex. Disruption of the eCB system by cannabis use during adolescence may therefore interfere with brain development such that adolescents are particularly susceptible to cannabis-related harms.^16^

Compared with non-using controls, adolescent cannabis users have poorer cognitive and executive functioning in some domains (for example, verbal and spatial working memory, attentional processes^17, 18^), alongside differing task-related neural responses (for example, greater BOLD response during response inhibition^19^ and spatial working memory tasks^20^), and morphological differences in medial temporal and frontal cortices^21^ and white matter integrity.^22, 23^ However, findings are mixed, limited by cross-sectional designs and small samples, and necessarily correlational in nature.^16^ Epidemiological findings further suggest that younger age of cannabis use onset may be associated with increased risk of addiction,^24, 25, 26, 27^ cognitive impairment^16, 28, 29^ and psychotic illness.^30, 31, 32^ Again such findings are limited since individuals starting use at a younger age will also typically have more cannabis exposures over a longer period of time, making it hard to dissociate the specific effect of age.

In rodents, repeated administration studies further suggest greater vulnerability to cannabis-related harm in adolescents. Adolescent exposure led to adulthood deficits in novel object recognition and spatial working memory, but not spatial learning.^9^ In adolescent rhesus monkeys Verrico *et al.*^33, 34^ found that both acute and repeated doses of THC led to impaired spatial but not object working memory; further, repeated THC prevented the maturational improvement in spatial working memory typically seen at that age, but did not affect the earlier developing object working memory. However, direct comparisons between adolescent and adult chronic exposure are scarce and findings have been inconsistent.^35, 36, 37, 38, 39, 40^

Evidence from acute administration studies in rats of increased adolescent vulnerability to the effects of cannabis is also mixed, with some suggesting acute cannabinoid treatment has a greater impairing effect on spatial and non-spatial learning (THC)^39, 40^ and object recognition (WIN55, 212-2)^41^ in adolescent compared with adult rats. Others however report the opposite, with evidence of greater acute impairments in adult rodents—including impaired novel object recognition (WIN55, 212-2)^38^ and spatial learning (WIN55, 212-2).^42^ Further, adult rats developed conditioned place (WIN55, 212-2)^43^ and taste (THC)^35^ aversion to cannabinoid treatment while adolescents did not, and adults produced more vocalizations when handled while intoxicated, suggesting greater drug-induced aversion.^35^ THC has also been found to have less anxiogenic^44^ or even anxiolytic^42^ effects, alongside reduced locomotor-suppression effects,^44^ in adolescent rats compared with adults. Translation of these findings to humans is limited by a number of factors, including the common use of potent synthetic cannabinoids with full CB1 receptor agonism rather than THC (for example, WIN55, 212-2), and often high doses compared with typical human consumption.

Despite mixed findings, cannabinoid administration studies in adolescent rodents and non-human primates predominantly suggest that the adolescent brain is differentially sensitive to the effects of cannabis. Should these findings translate to humans, these age-related sensitivities may contribute to an increased risk of cannabis-related harms in teenagers. Indeed, it has been suggested that if adolescents are less sensitive to the acute negative effects (for example, increased anxiety) of cannabis (and other recreational substances, as has been suggested for alcohol^45^) then this may lead to greater drug consumption than adults.^44^ However, acute studies in humans have rarely explored the influence of age on drug effects. Indeed, we are aware of no controlled studies in which cannabis was administered to individuals under 18 years of age.

The present study therefore aimed to compare the acute effects of cannabis in adolescent and adult users. In adults, acute cannabis administration typically induces episodic memory impairments^46, 47^ and may impair working memory and response inhibition.^48, 49^ Acutely cannabis also increases subjective drug-related experiences (for example, feeling ‘stoned'), and psychotomimetic symptoms.^50, 51^ On the basis of preclinical findings, we hypothesized that adolescents would be less sensitive to the intoxicating^35, 43, 44^ and anxiogenic^42, 44^ effects of cannabis compared with adults. Further, given links between earlier onset of cannabis use and psychosis,^30, 31, 32^ we predicted more psychotomimetic effects of cannabis in adolescents than adults. Finally, we hypothesized greater cognitive impairment following cannabis in adolescents than adults,^39, 40, 41^ as indexed by spatial working memory, episodic memory and response inhibition.

## Methods

### Design and participants

A mixed within- and between-subjects, double-blind, cross-over design was used to compare the acute effects of active and placebo cannabis on adolescents and adults. Treatment order was counterbalanced for task version and randomized via random number generator within each age group.

We recruited 20 adolescent (aged 16–17 years) and 20 adult (24–28 years) male cannabis users, via local and online (social media) advertising and word-of-mouth. The following inclusion criteria were assessed at telephone screening: male gender (due to evidence of sex differences in onset of puberty and ontogeny of adolescent brain development); current cannabis use between 1 and 3 days per week; at least 6 months of regular (at least once per week) cannabis use; no extended period (>1 month) of daily use; score ⩽3 on the Cannabis Severity of Dependence Scale reflecting the validated adolescent cut-off for dependence;^52^ no other illicit drug was used more than twice per month; no current mental health problem or history (personal or immediate family) of psychosis-related disorders; healthy-range body mass index and blood pressure (BP). Participants were asked to remain abstinent from all drugs including alcohol but not cigarettes for 24 h before each testing session.

The study was approved by UCL Research Ethics Committee. All participants provided written informed consent (in the UK 16–17-year olds are able to provide informed consent without additional parental consent or assent). Participants were reimbursed for their time (£7.50 per hour) and travel expenses.

### Drug administration

Medicinal-grade active (Bedrobinol; THC 12.0%) and placebo (THC <0.3%) cannabis were imported under UK Home Office license from Bedrocan (Veendan, The Netherlands). Dose was weight-adjusted as age differences in body weight were anticipated. Following previous protocols,^53, 54, 55^ participants received 0.89 mg kg^−1^ of cannabis, corresponding to ~8.0 mg THC for an individual weighing 75 kg. This dose corresponds to that contained in about a third of a typical joint.^56^ Similar doses have previously been shown to produce robust subjective effects via the administration method used in this study.^53, 54, 55^

Drug was administered via a Volcano Medic vaporizer (Storz and Bickel, Tuttlingen, Germany), operating at 210 °C. This method has been shown to be safe, producing equivalent pulmonary and plasma cannabinoid levels to those from smoked cannabis, but with lower expired carbon monoxide levels.^57, 58, 59^ Vapor was collected in a ‘balloon' with a non-return valve, and inhaled according to a previous timed breath-holding protocol.^55^ Participants inhaled, held their breath for 8 s and repeated this at their own pace until the balloon was empty. Each dose was vaporized in two sequentially administered balloons to minimize residual cannabinoids.

### Measures

#### Baseline assessments

Premorbid verbal intelligence was assessed by the Wechsler Test of Adult Reading,^60^ and scores were adjusted for age. Depression and anxiety were assessed on the Beck Depression Inventory^61^ and Beck Anxiety Inventory.^62^ A validated short version of the UPPS-P Impulsive Behaviour Scale (SUPPS-P)^63, 64^ indexed impulsivity and the Schizotypal Personality Questionnaire^65^ indexed schizotypy.

#### Drug use

A structured interview recorded: lifetime use (yes/no); time since last use (days); duration of use (years); frequency (days/month); and amount per session (alcohol units (standard UK units of alcohol; equivalent to 8 g of pure alcohol or ~3/5ths of a NIAAA standardized drink) per typical drinking session; cigarettes/day; other illicit drugs grams/pills/tabs). Instant urine drug screens at the start of every session assessed recent use of illicit drugs (amphetamine, barbiturates, benzodiazepines, cocaine, MDMA, methamphetamine, methadone, opiates, oxycodone, phencyclidine (Supplementary Table S1).^66^ Problematic drug use was assessed using the Cannabis Abuse Screening Test,^67^ the Fagerstrom Test for Nicotine Dependence^68^ and the Alcohol Use Disorders Identification Test.^69^

#### Physiological measurements

Body weight, BP and heart rate were measured at baseline. BP and heart rate were monitored throughout drug administration sessions.

#### Subjective ratings

Participants provided ratings from 0 (not at all) to 10 (extremely) for ‘Stoned', ‘High', ‘Feel drug effect', ‘Like drug effect' ‘Alert', ‘Anxious', ‘Paranoid', ‘Dry mouth', ‘Enhanced color perception', ‘Enhanced sound perception', ‘Want to have food' and ‘Want to have cannabis', at −6 min (apart from ‘Feel drug effect' and ‘Like drug effect'), +7 min, +34 min and +77 min (drug administration started at 0 min).

#### Psychotic-like symptoms

Participants completed the Psychotomimetic States Inventory (PSI), a self-report questionnaire sensitive to the acute psychotomimetic effects of cannabis.^70, 71^

#### Memory tasks

##### Prose recall

This episodic memory task was adapted from the Rivermead Behavioural Memory Test battery.^72^ Participants listened to a 30 s story and then for 1 min wrote down what they remembered immediately and again after ~1 h. Each story contained 21 ‘idea units' and scoring was systematic.

##### Spatial N-back

A computerized spatial version of the N-back task^73, 74^ was used to assess spatial working memory. Stimuli appeared sequentially in one of the six possible locations on screen, around a fixation cross. Participants responded ‘yes' or ‘no' as to whether the stimulus was in the same position as the stimulus one before (low load; ‘1-back') or two before (high load; ‘2-back'). Performance was indexed by discriminability (*d*') and reaction time for correct trials.

#### Response inhibition

##### Stop signal

A staircase tracking version of the stop signal was used to measure response inhibition.^75^ Stimuli (white arrows) appeared sequentially in the center of the screen; participants responded when the white arrow pointed left or right by pressing either the left or right arrow key. On 25% of trials, the arrow became blue following a variable delay (signal trials); on these trials participants were instructed to not press either arrow key (that is, inhibit the prepotent response). Performance was assessed with stop-signal reaction time and accuracy on no-signal trials.

### Procedure

Following screening, participants attended a 1-h baseline session during which they provided informed consent, completed baseline measures, drug histories, problematic use questionnaires, task training and physiological measurements.

Participants then completed two test sessions separated by at least 7 days. Participants first provided baseline subjective ratings, and BP and heart rate were measured (Time 1; T1). Active or placebo cannabis was then administered and participants again completed subjective ratings, BP and heart rate measures (Time 2; T2). Tasks and state questionnaires were then completed in the following order; prose recall (immediate), PSI, subjective ratings (Time 3; T3), spatial N-back, stop signal, prose recall (delayed), subjective ratings (Time 4; T4), BP and heart rate (T4). Test sessions finished 80 min after drug inhalation.

### Power calculation

To detect a medium effect size (*f*=0.25) for the key interaction of interest (group × drug), with 80% power at an alpha of 5%, we required a sample size of 34. To account for drop-out and task adherence issues, we tested 40 in total.

### Statistical analysis

All analyses were conducted with SPSS 21.0. Syntax and data are available from CM. Outliers and normality were assessed via diagnostic plots for all analyses. Extreme outliers (>3 times interquartile range) were winsorized within-group. Greenhouse–Geisser corrections were applied for violations of sphericity. Independent *t*-test, chi-squared or Mann–Whitney analyses were conducted as appropriate to compare groups (adolescent, adult) on demographic and baseline measures.

Mixed analysis of variance was conducted for all test outcomes, with the between-subjects factor of group (adolescent, adult; coded as 1, 2, respectively) and within-subjects factor of drug (placebo, cannabis; coded as 1, 2, respectively). Additional within-subjects factors were included for relevant analyses: time (T1, T2, T4; coded as 1, 2, 3, respectively) for physiological data; time (T1–T4; coded as 1, 2, 3, 4, respectively) for subjective ratings (only T2–T4 (coded as 1, 2, 3, respectively) were analyzed for stoned (due to floor effects), feel drug effect and like drug effect (as these were not collected at T1)); PSI subscale (thought distortion, perceptual distortion, cognitive disorganisation, anhedonia, manic experience; coded as 1, 2, 3, 4, 5, respectively; paranoia subscale was not included in analyses due to floor effects); N-back memory load (low, high; coded as 1, 2, respectively); prose recall delay (immediate, delayed; coded as 1, 2, respectively). Main effects and interactions with time were tested and explored via Helmert contrasts (comparing 'Pre-drug' (T1) with 'Post-drug' (mean of T2–T4)), to reduce the number of comparisons. Other interactions were explored via pairwise comparisons with local Bonferroni correction. Drug order was added as an additional between-subjects factor (placebo-first, cannabis-first; coded as 1, 2, respectively) and results were compared with reported primary analyses; unless otherwise noted results were unaffected by drug order. All statistical tests were two-tailed. Supplementary Table S2 contains descriptive data for memory and inhibition tasks.

## Results

### Demographics

Adolescents were younger, and had lower body weight. Groups did not differ on verbal IQ, Beck Anxiety Inventory, Beck Depression Inventory, SUPPS-P or Schizotypal Personality Questionnaire (Table 1). Adolescents currently used cannabis for more days per month than the adults, and the age of first cannabis use was younger for the adolescents compared with the adults, but overall the adults had used for longer. Groups did not differ on Cannabis Abuse Screening Test score, time since last cannabis use, or likelihood of a positive THC urine screen at baseline.

### Physiological data

#### Heart rate

**Figure 1:**
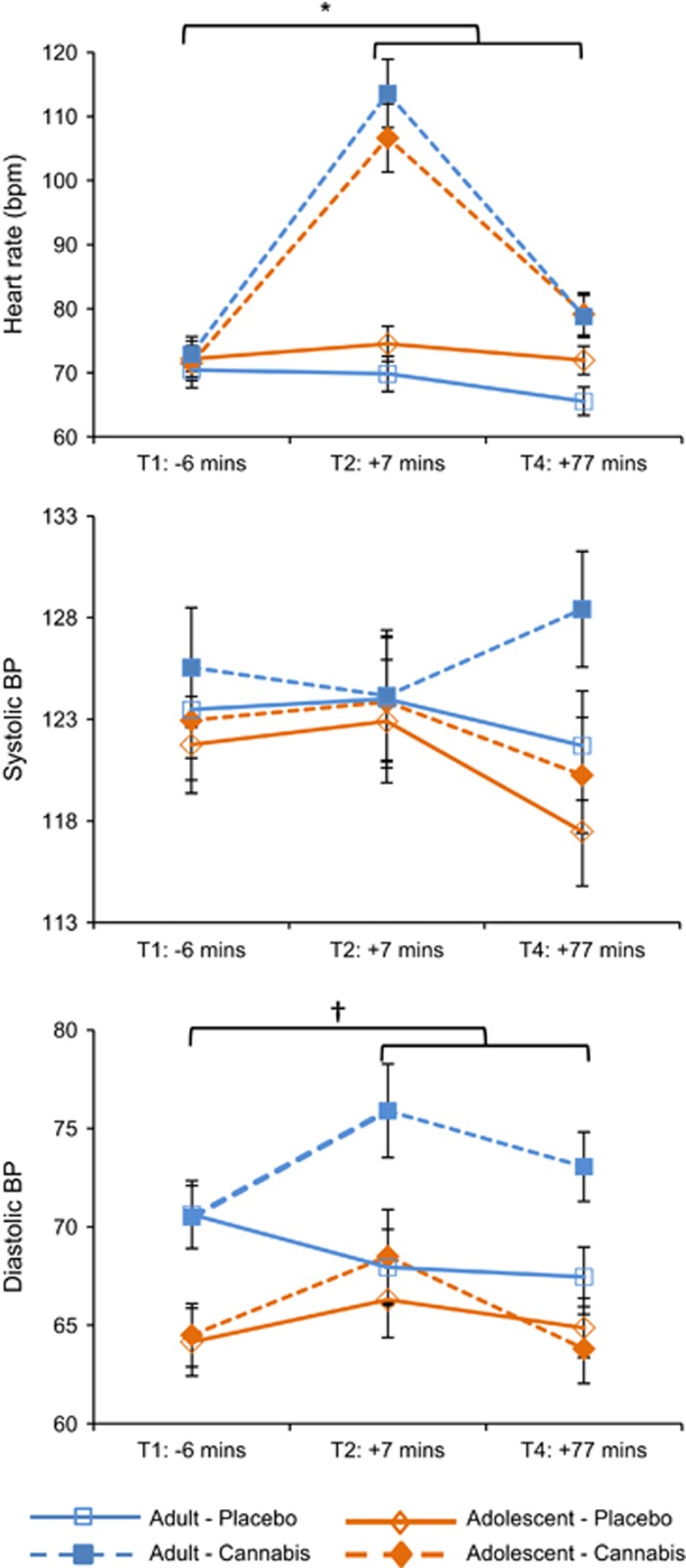
Mean (s.e.) values for heart rate (bpm), systolic and diastolic blood pressure (BP) for adolescents and adults on cannabis and placebo. *Heart rate increased from Pre-drug to Post-drug for cannabis (*P*<0.001) but not placebo (*P*=0.449); †=for adults diastolic BP increased from Pre-drug to Post-drug on cannabis (*P*=0.016) but not placebo (*P*=0.060).

An interaction of drug × time (F_1,38_=82.879, *P*<0.001, *η*^2^*p*=0.69) was found, with heart rate increasing from Pre-drug to Post-drug for cannabis (*P*<0.001, *η*^2^*p*=0.65) but not placebo (*P*=0.449, *η*^2^*p*=0.01; Figure 1). Main effects of drug (F_1,38_=89.327, *P*<0.001, *η*^2^*p*=0.70) and time (F_1,38_=44.141, *P*<0.001, *η*^2^*p*=0.54) also emerged.

#### Systolic BP

No main effects or interactions were found.

#### Diastolic BP

Interactions of drug × group × time (F_1,38_=4.393, *P*=0.043, *η*^2^*p*=0.10), drug × group (F_1,38_=4.744, *P*=0.036, *η*^2^*p*=0.11) and drug × time (F_1,38_=4.977, *P*=0.032, *η*^2^*p*=0.12) emerged. For adolescents, there was no drug × time interaction (*P*=0.919, *η*^2^*p*<0.01); while for adults a drug × time interaction (*P*=0.010, *η*^2^*p*=0.30) revealed an increase in diastolic BP from Pre-drug to Post-drug for cannabis (*P*=0.016, *η*^2^*p*=0.27), but no change over time for placebo (*P*=0.060, *η*^2^*p*=0.17). Main effects of drug (F_1,38_=7.390, *P*=0.010, *η*^2^*p*=0.16) and group (F_1,38_=7.998, *P*=0.007, *η*^2^*p*=0.17) also emerged.

### Subjective ratings

#### Stoned

**Figure 2:**
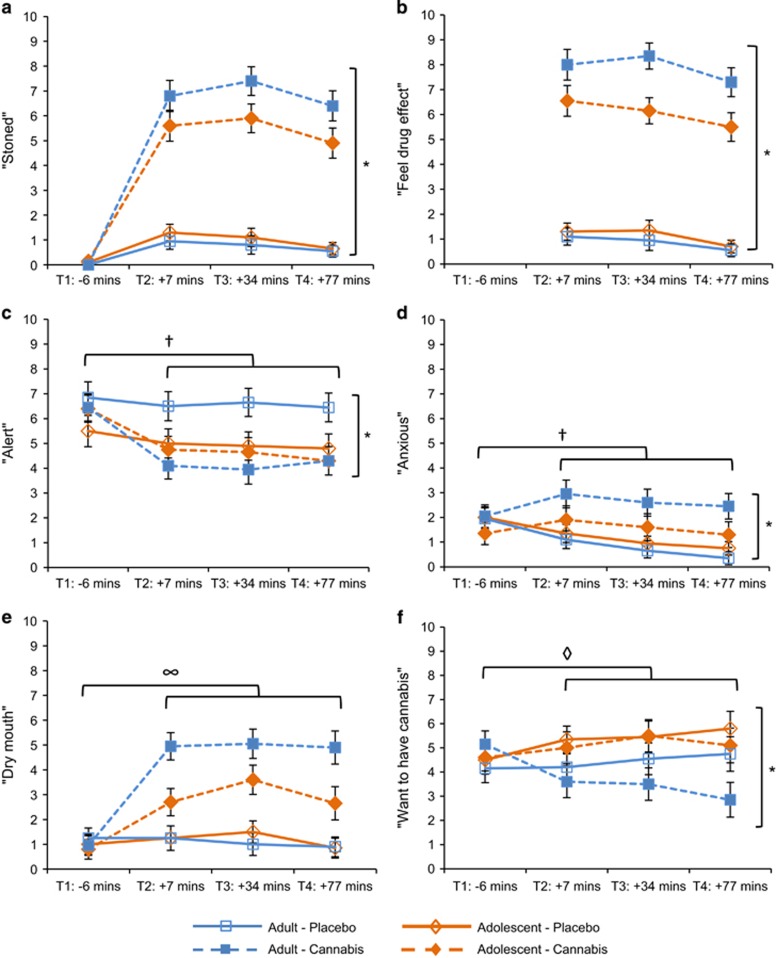
Mean (s.e.) values for subjective ratings (0–10) for ‘stoned', ‘feel drug effect', ‘alert', ‘anxious', ‘dry mouth', ‘want to have cannabis', for adolescents and adults on placebo and cannabis. *Drug × group interaction (*P*⩽0.046); ^†^drug × time interaction (*P*⩽0.003); ^∞^drug × group × time interaction (*P*=0.004); ^◊^group × time interaction (*P*=0.004).

There was an interaction of drug × group (F_1,38_=4.893, *P*=0.033, *η*^2^*p*=0.11; Figure 2). Ratings of both adolescents (*P*<0.001, *η*^2^*p*=0.65) and adults (*P*<0.001, *η*^2^*p*=0.78) were higher after cannabis compared with placebo; however, the increase was larger in adults. Main effects of drug (F_1,38_=200.055, *P*<0.001, *η*^2^*p*=0.84) and time (F_2,63_=8.271, *P*=0.001, *η*^2^*p*=0.18) also emerged.

#### Feel drug effect

There was an interaction of drug × group (F_1,38_=8.877, *P*=0.005, *η*^2^*p*=0.19), with adolescents feeling the drug effect less than adults after cannabis (*P*=0.017, *η*^2^*p*=0.14), but not after placebo (*P*=0.565, *η*^2^*p*=0.01). Main effects of drug (F_1,38_=297.629, *P*<0.001, *η*^2^*p*=0.89) and time (F_2,65_=9.629, *P*<0.001, *η*^2^*p*=0.20) also emerged.

#### Alert

There was an interaction of drug × group (F_1,38_=9.123, *P*=0.004, *η*^2^*p*=0.19), with adolescents rating no difference in alertness on cannabis compared with placebo (*P*=0.955, *η*^2^*p*<0.01), whereas adults rated lower alertness on cannabis compared with placebo (*P*<0.001, *η*^2^*p*=0.33). There was also an interaction of drug × time (F_1,38_=42.844, *P*<0.001, *η*^2^*p*=0.53); with alertness decreasing from Pre-drug to Post-drug in both sessions, though the decrease was larger for cannabis (*P*<0.001, *η*^2^*p*=0.65) than for placebo (*P*=0.005, *η*^2^*p*=0.19). Main effects of drug (F_1,38_=9.613, *P*=0.004, *η*^2^*p*=0.20) and time (F_1,38_=60.071, *P*<0.001, *η*^2^*p*=0.61) also emerged.

#### Anxious

There was an interaction of drug × group (F_1,38_=4.272, *P*=0.046, *η*^2^*p*=0.10), with adolescents reporting no difference in anxiety between drugs (*P*=0.516, *η*^2^*p*=0.01), but adults reporting more anxiety on cannabis compared with placebo (*P*=0.001, *η*^2^*p*=0.25). There was also an interaction of drug × time (F_1,38_=9.914, *P*=0.003, *η*^2^*p*=0.21); with no change over time in anxiety for cannabis (*P*=0.275, *η*^2^*p*=0.03) and a decrease in anxiety from Pre-drug to Post-drug for placebo (*P*<0.001, *η*^2^*p*=0.39). A main effect of drug (F_1,38_=8.969, *P*=0.005, *η*^2^*p*=0.19) also emerged.

#### Dry mouth

There were interactions of drug × group × time (F_1,38_=9.417, *P*=0.004, *η*^2^*p*=0.20), drug × group (F_1,38_=6.436, *P*=0.015, *η*^2^*p*=0.15) and drug × time (F_1,38_=72.572, *P*<0.001, *η*^2^*p*=0.66). Both adolescents (*P*<0.001, *η*^2^*p*=0.52) and adults (*P*<0.001, *η*^2^*p*=0.72) reported an increase in dry mouth from Pre-drug to Post-drug on cannabis, though the increase was greater for adults. On placebo there was no change in dry mouth over time for adolescents (*P*=0.495, *η*^2^*p*=0.03) or adults (*P*=0.244, *η*^2^*p*=0.07). Main effects of drug (F_1,38_=44.682, *P*<0.001, *η*^2^*p*=0.54) and time (F_1,38_=46.168, *P*<0.001, *η*^2^*p*=0.55) also emerged.

#### Want to have cannabis

There was an interaction of group × time (F_1,38_=9.661, *P*=0.004, *η*^2^*p*=0.20). From Pre-drug to Post-drug, wanting of cannabis increased in the adolescents (*P*=0.048, *η*^2^*p*=0.19) and decreased in the adults (*P*=0.031, *η*^2^*p*=0.22). There was also an interaction of drug × time (F_1,38_=5.933, *P*=0.020, *η*^2^*p*=0.14); wanting of cannabis increased after taking placebo (*P*=0.037, *η*^2^*p*=0.11), but did not change after taking cannabis (*P*=0.177, *η*^2^*p*=0.05).

#### Other subjective ratings

Comparable analyses revealed that compared with placebo, cannabis increased subjective ratings for ‘paranoid', ‘mentally impaired', ‘high', ‘like drug effect', ‘want to have food', ‘enhanced color perception' and ‘enhanced sound perception' (all *P*'s <0.05). However, there were no group-related differences or interactions for any of these ratings (all p's >0.05).

### Psychotomimetic effects

#### PSI

**Figure 3:**
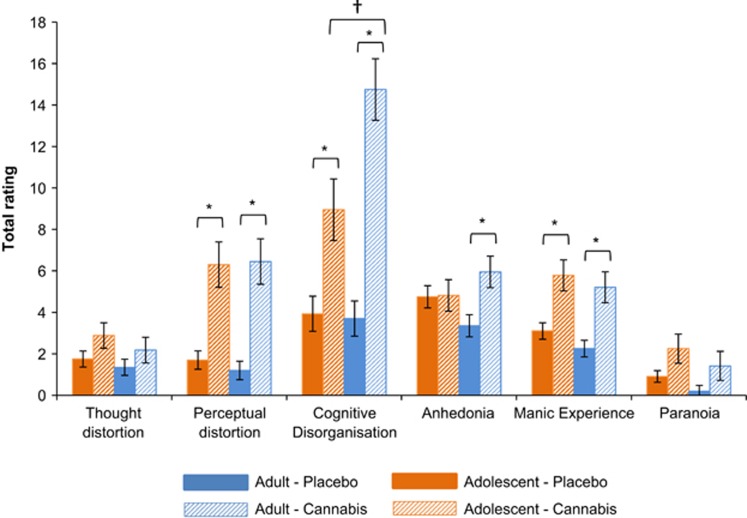
Mean (s.e.) values for total ratings of each subscale of the Psychotomimetic States Inventory (PSI), for adolescents and adults on placebo and cannabis. *Ratings on cannabis were higher than on placebo (*P*⩽0.001); ^†^ratings on cannabis were higher for adults than adolescents (*P*=0.009).

There were interactions of drug × subscale × group (F_4,152_=6.241, *P*<0.001, *η*^2^*p*=0.14), subscale × group (F_4,152_=5.111, *P*=0.001, *η*^2^*p*=0.12), drug × subscale (F_3,116_=32.032, *P*<0.001, *η*^2^*p*=0.46), and drug × group (F_1,38_=4.281, *P*=0.045, *η*^2^*p*=0.10; Figure 3). Neither group had increased thought distortion following cannabis compared to placebo (all *P*'s⩾0.076, all *η*^2^*p*⩽0.08). Both groups had higher perceptual distortion, manic experience and cognitive disorganization ratings on cannabis compared with placebo (all *P*'s⩽0.001, all *η*^2^*p*⩾0.27). On cannabis adults reported higher cognitive disorganization than adolescents (*P*=0.009, *η*^2^*p*=0.17). Lastly, cannabis increased anhedonia in adults (*P*=0.001, *η*^2^*p*=0.25) but not adolescents (*P*=0.925, *η*^2^*p*< 0.01). Main effects of drug (F_1,38_=66.453, *P*<0.001, *η*^2^*p*=0.64) and subscale (F_3,102_=43.544, *P*<.001, *η*^2^*p*=0.53) also emerged.

### Cognitive tasks

#### Spatial N-back

Five participants were excluded (three adults, two adolescents) due to <50% accuracy.

##### Discriminability

Main effects of drug (F_1,33_=30.495, *P*<0.001, *η*^2^*p*=0.48) and load (F_1,33_=26.054, *P*<0.001, *η*^2^*p*=0.44) were found. Discriminability was poorer on cannabis (M=2.47, s.e.=0.12) than placebo (M=3.09, s.e.=0.10) and on high load (M=2.49, SE=0.13) than low load (M=3.07, s.e.=0.09).

##### Reaction time (correct trials)

Initial analyses demonstrated main effects of drug (F_1,33_=12.221, *P*=0.001, *η*^2^*p*=0.27) and load (F_1,33_=44.430, *P*<0.001, *η*^2^*p*=0.57), with no interactions. Reaction times were longer on cannabis than placebo and on high load (M=706.77, s.e.=25.58) than low load (M=566.95, s.e.=16.87). However, after adding drug order to the model, an interaction of drug × group (F_1,31_=4.447, *P*=0.043, *η*^2^*p*=0.13) also emerged. For adolescents there was no difference in reaction times between cannabis (M=632.63, s.e.=30.74) and placebo (M=589.75, s.e.=23.83; *P*=0.076, *η*^2^*p*=0.10), while for adults reaction times were longer after cannabis (M=720.40, s.e.=32.14) than placebo (M=606.31, s.e.=24.92; *P*<0.001, *η*^2^*p*=0.41).

#### Prose recall

**Figure 4:**
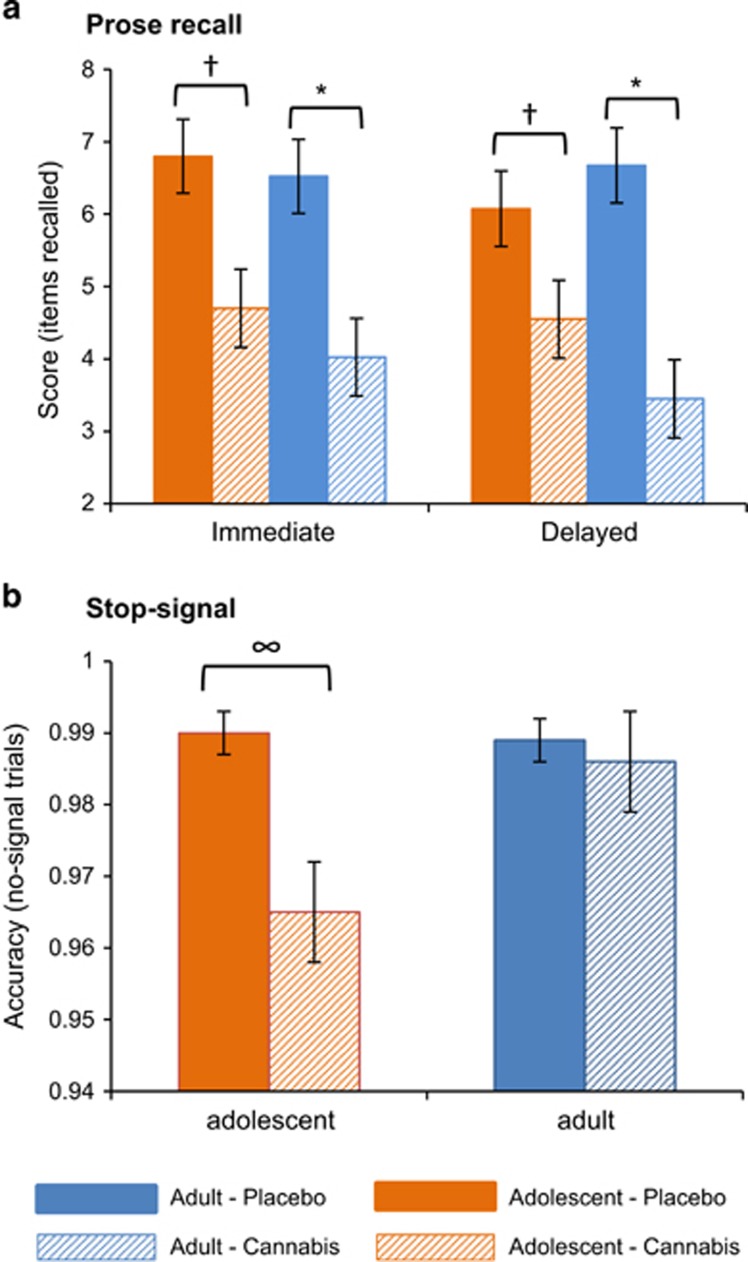
Mean (s.e.) values for (**a**) prose recall score (number of items recalled, out of a total of 21) and (**b**) stop-signal accuracy (proportion of no-signal trials with a correct response), for adolescents and adults on placebo and cannabis. *Adult scores after taking cannabis were lower than after taking placebo (*P*<0.001); ^†^adolescent scores after taking cannabis were lower than after taking placebo (*P*⩽0.038); ^∞^adolescents were less accurate after taking cannabis than placebo (*P*=0.001).

There was an interaction of drug × delay × group (F_1,38_=5.518, *P*=0.024, *η*^2^*p*=0.13), with adolescents recalling fewer items after cannabis than placebo, both immediately (*P*=0.002, *η*^2^*p*=0.22) and after the delay (*P*=0.038, *η*^2^*p*=0.11; Figure 4a). Adults also recalled fewer items after cannabis than placebo, both immediately (*P*<0.001, *η*^2^*p*=0.28) and after the delay (*P*<0.001, *η*^2^*p*=0.35); however, the reduction in items recalled after cannabis compared with placebo for delayed recall was twice as large in adults than adolescents. A main effect of drug (F_1,38_=25.869, *P*<0.001, *η*^2^*p*=0.41) also emerged.

#### Stop-signal

Two participants (one adult, one adolescent) had missing data due to technical issues; one adult was excluded due to an improbable stop-signal reaction time (<50 ms^[ref^. ^76^^]^).

##### Stop-signal reaction time

No main effects or interactions were found.

##### Accuracy on no-signal trials

There was an interaction of drug × group (F_1,35_=4.906, *P*=0.033, *η*^2^*p*=0.12), with adolescents being less accurate on cannabis compared with placebo (*P*=0.001, *η*^2^*p*=0.28), whereas drug did not affect adults' accuracy (*P*=0.644, *η*^2^*p*=0.01; Figure 4b). A main effect of drug (F_1,35_=8.306, *P*=0.007, *η*^2^*p*=0.19) also emerged.

### Correlations

Within-group correlations were conducted between all cannabis session outcomes in which we found group main effects or interactions, and variables showing baseline group differences (at *P*<0.10; Table 1), including administered cannabis weight. Cannabis weight was not found to correlate with any outcome in either group. None were found to correlate (at *P*<0.10) with any outcome measure in both the adolescent and adult groups, and so were not entered into models.

## Discussion

In what we believe is the first study to examine the causal effects of acute cannabis administration in human adolescence and adulthood, we found two differing profiles of effects. Compared with adults, adolescents experienced blunted subjective, physiological and psychotomimetic effects of cannabis, while cannabis impaired inhibitory processes in adolescents but not adults. Specifically, on cannabis adolescents reported feeling less stoned, feeling less effect of the drug, less dry mouth and less cognitive disorganization than adults. The adults were also markedly more anxious and less alert during the cannabis session than the placebo session, while no session difference was found for the adolescents (however, since these group differences did not differ over time, these may be session effects rather than effects of cannabis). Indeed, there was no subjective rating on which adolescents reported greater drug effect than adults. Further, adults' but not adolescents' diastolic BP rose after cannabis.

Intriguingly, we found opposing effects between age groups on wanting of cannabis following drug administration. The adolescents did not show a typical satiety effect, wanting more cannabis post drug regardless of whether they had taken cannabis or placebo. Meanwhile the adults wanted less cannabis post drug, an effect that appears to be driven by a decrease in wanting following cannabis but not after placebo (although this putative interpretation remains tentative in the absence of a group × drug × time interaction).

In terms of cognitive effects, when intoxicated with cannabis adults showed greater impaired recall of prose following a delay than adolescents. After adjusting for drug order, the adults also had longer response times on the spatial working memory task following cannabis, while the adolescents were not affected. Although neither group was impaired at inhibiting a pre-potent response following cannabis, the adolescents but not adults were less accurate on the inhibition task after cannabis.

These results are in line with our first hypothesis that adolescents would be less sensitive to physiological, intoxication and anxiogenic effects compared with adults. These findings accord with the preclinical evidence that shows reduced anxiogenic, aversive and locomotor effects in adolescent rodents.^35, 42, 43, 44^ Further, while our second hypothesis predicted a greater degree of psychotomimetic effects following cannabis in the adolescents compared with the adults, we instead found the opposite: cognitive disorganization was especially elevated in adults compared with adolescents after cannabis. This unexpected finding is however in agreement with our first hypothesis of lesser intoxication effects in adolescents, perhaps suggesting a common mechanism by which adolescents are resilient to the acute negative effects of cannabis. It may also reflect an awareness in adults of the greater cognitive impairments they were experiencing, rather than amplified psychotic-like effects of cannabis per se. We also found that cannabis increased anhedonia symptoms in adults but not in adolescents; interestingly however, on placebo the adolescents had (non-significantly) higher levels of anhedonia than the adults.

Lastly, partial support for our third hypothesis, that we would see greater cognitive impairment following cannabis in adolescents than adults, was seen in greater impairment of response inhibition accuracy following cannabis in the adolescents compared with adults. However, contrary to expectations we did not see greater cannabis-related memory impairment in the adolescents, instead finding evidence of greater impairment in adults. Preclinical evidence for greater adolescent sensitivity to acute memory-impairing effects of cannabis is however inconsistent.^77^ In adult humans cannabis appears to selectively impair episodic and working memory domains,^78^ leaving other memory domains intact, while rodents typically become impaired on a wide range of memory tasks across domains including object recognition and spatial learning, implying that preclinical findings for cannabis and memory may be somewhat limited in translation.

These findings have important implications for public health, especially given the current changes in legislation that are making cannabis more available and may influence adolescent use in several parts of the globe. If adolescents do not feel satiated after an acute dose of the drug while also experiencing fewer negative effects, they may well use more cannabis in a smoking session than adults,^44^ potentially contributing to the increased risk of long-term harms associated with younger age of use, including addiction.^16^ In turn, adults' experience of more negative effects of cannabis may limit their use and reduce their risk of harms, which would concur with the declining prevalence of cannabis use seen from early adulthood.^4^ A clear next step from these findings is therefore replication (importantly with females as well as males) and then assessment of naturalistic use of cannabis in different age groups, using measures that clearly record weight and potency of cannabis smoked,^79^ topography of inhalation,^56^ alongside ratings of subjective negative and positive intoxication effects. Tracking these participants longitudinally would be important in determining how these age-related sensitivities may impact in the long term on cannabis use patterns and mental and physical health outcomes.

Our study has several critical strengths. Importantly our groups were well matched on baseline measures including premorbid IQ and levels of anxiety, depression, impulsivity and schizotypy. This increases our confidence that participants in the two age groups were drawn from similar populations, and maximizes comparability between groups. Further the use of cannabis plant material, rather than extracted or synthetic cannabinoids, via an ecologically valid administration procedure (that is, inhalation) enhances the relevance of our findings to the real world use of this drug. Administering a known THC dosage that closely corresponds to that contained in about a third of a typical joint,^56^ which was weight adjusted to allow for weight differences in adolescents and adults, are both strengths of this controlled study.

The study is not without limitations. First, we cannot speak to mechanism of the reported age-related sensitivities. Although the findings may represent age-related neural sensitivities to cannabis, there are a number of alternative explanations. Adolescents have a higher basal metabolism than adults,^80, 81^ alongside lower percentage body fat,^82, 83^ potentially affecting the speed of THC metabolism between the groups. Should THC and its by-products be metabolized more quickly in adolescents than adults, this could potentially result in the reduced subjective and episodic memory effects seen in adolescents; however, if drug metabolism in the adolescents was faster, a quicker decline of drug effects would be expected, which does not appear to be the case. Further, this would not explain the adolescent's impaired inhibition accuracy when the adults were unaffected. Group differences in the effect of cannabis on diastolic BP are also intriguing, though adolescents' diastolic BP was lower on both sessions at baseline, consistent with normative data.^84^ This finding should also be viewed alongside a lack of a group difference in the more robust effect of cannabis increasing heart rate. Relatedly, participants were given a weight-adjusted dose, meaning that because adolescents typically weigh less than adults,^85^ on average they received a lower dose. We cannot therefore rule out the possibility that the blunted effects seen in the adolescents are due to the reduced dose; however, again this would not explain the overall pattern of results including the adolescents' (but not adults') impaired response inhibition accuracy. Moreover, critically the weight of cannabis administered did not correlate with any outcome in either group. Groups could potentially be matched for body weight in future research, however this would result in biased samples that do not reflect the population as a whole. An important goal now is to investigate the mechanisms by which these apparent group differences occur, for instance, a first step would be to repeat key components of our protocol using an fMRI paradigm.

Second, all our participants were necessarily regular cannabis users, raising the possibility that our findings may be affected by group differences in past cannabis use. Although the groups were matched for cannabis abuse symptomology and days since last use, the adolescents did report more days of cannabis use per month than the adults (11 days versus 8 days); further while the adults had been using for more years, they had started using from an older age. Tolerance to some cannabis effects following frequent use has been reported (including for spatial working memory and episodic memory^78^), however findings are inconsistent^86^ and little is known about the development of cannabis tolerance and how different usage patterns affect this. As such it is possible that differing cannabis use histories and patterns may explain group differences in outcomes. Importantly however, none of our measures of cannabis use correlated with outcomes in both the adolescent and adult groups. Relatedly, the adolescents were more frequent and heavier cigarette smokers, with higher nicotine-dependence scores, and they had started tobacco smoking from a younger age than the adults. The groups were well matched for age of first alcohol use, but the adolescents were less frequent alcohol drinkers. It is possible that cross-tolerance to cannabis from previous alcohol or tobacco use may occur, though we are not aware of evidence demonstrating such an effect. A recent ecological momentary assessment study suggested that acutely tobacco use may offset acute impairment of working memory from cannabis,^87^ though this has yet to be replicated in a controlled study. It is possible therefore that the age group differences in alcohol and cigarette use may be contributing to our findings.

Third, we recruited only males, due to differing age of puberty onset and potentially differing brain development trajectories between sexes, thus precluding generalization of findings to teenage girls. Samples in cannabis research are often predominantly male and gender effects have rarely been assessed, with inconsistent findings.^78^ Some have shown heightened subjective^88^ and working memory^89^ effects in women compared with men, though others found no differences.^90^ Recently it was found that younger age of cannabis use onset predicted poorer episodic memory in women but not men,^91^ suggesting that there may be age-dependent sex differences in the cognitive effects of cannabis. Given such findings, there is a clear evidence gap regarding the effects of cannabis in young women and girls and future research should assess whether our findings generalize to females.

Finally, since this was a novel study, with multiple statistical comparisons and limited or mixed evidence on which to base our prior hypotheses, it is important to treat these findings with caution. Replications with larger sample sizes (which can now be determined according to effect sizes reported in this paper) are required before strong conclusions can be drawn.

In conclusion, compared with adults, adolescent cannabis users experienced blunted subjective, physiological and memory impairing effects of cannabis. Further, adolescents were not satiated by cannabis and the drug impaired their inhibitory processes while leaving those of adults intact. To our knowledge, this is the first study to administer cannabis in a controlled setting to humans under 18, and it therefore represents a significant step forward in the translation of preclinical developmental psychopharmacology. In agreement with preclinical cannabinoid administration studies, we found evidence to suggest that human adolescents and adults are differentially sensitive to the acute effects of cannabis. Longitudinal research is now needed to determine the degree to which age-related sensitivities are indeed contributing to escalated use and increased risk of cannabis-related harms in adolescent cannabis users.

## Figures and Tables

**Table 1:** Demographic and baseline variables for adolescents and adults

	*Adolescents (n=20)*	*Adults (n=20)*	*Test statistic*	P*-value*
	*Mean (s.d.)*	*Mean (s.d.)*		
*Demographics*				
Age (years)	17.08 (0.44)	25.49 (1.07)	*U*=400.000	<0.001^*^
Body weight (kg)	66.40 (10.30)	74.96 (10.12)	*U*=296.000	0.009^*^
Cannabis weight (mg)	58.90 (7.65)	65.44 (6.56)	*U*=299.500	0.006*
Verbal IQ (*n=*39)	110.20 (11.29)	115.11 (8.70)	*U*=245.000	0.127
				
*Baseline questionnaires*				
Beck Anxiety Inventory	4.55 (4.62)	6.45 (7.09)	*U*=234.500	0.355
Beck Depression Inventory	6.35 (4.66)	4.55 (4.38)	*U*=152.000	0.201
SUPPS-P Impulsive Behaviour Scale	45.55 (8.00)	45.40 (5.94)	*t*_38_=0.067	0.947
Schizotypal Personality Questionnaire	20.90 (10.90)	15.21 (11.24)	*U*=145.000	0.142
				
*Cannabis use*				
Age first tried cannabis (years)	14.73 (1.25)	17.71 (3.00)	*U*=338.000	<0.001^*^
Last used cannabis (days)	3.35 (2.52)	4.75 (3.78)	*U*=259.500	0.108
Duration of cannabis use (years)	2.35 (1.24)	7.78 (2.85)	*U*=378.500	<0.001^*^
Cannabis use frequency (days per month)	10.58 (4.33)	7.94 (5.27)	*U*=121.000	0.033*
Positive THC urine at baseline (*n=*37); %(*n*)	83.33 (15)	63.16 (12)	*χ*^2^_1_=1.908	0.167
Cannabis Abuse Screening Test	6.45 (2.72)	5.60 (3.56)	*t*_38_=0.848	0.402
				
*Cigarette use*				
Ever used cigarettes*;* %(*n*)	95.00 (19)	75.00 (15)	*χ*^2^_1_=3.137	0.077
Age first tried cigarettes (years)^b^	15.06 (1.49)	17.21 (2.61)	*U*=279.000	0.003^*^
Duration of cigarette use (years)	1.91 (1.41)	7.60 (3.44)	*U*=356.500	<0.001^*^
Cigarette use frequency (days per month)	19.28 (12.36)	10.37 (11.62)	*U*=120.500	0.030^*^
Cigarettes per day	3.74 (2.83)	1.84 (2.06)	*U*=107.500	0.011^*^
Fagerström Test for Nicotine Dependence	1.30 (1.03)	0.20 (0.70)	*U*=81.000	<0.001^*^
Carbon monoxide at baseline (p.p.m.; *n=*38)	6.00 (4.55)	5.68 (3.96)	*U*=163.000	0.624
				
*Alcohol use*				
Ever used alcohol; %(*n*)	100.00 (20)	100.00 (20)	NA	NA
Age first tried alcohol (years)	14.07 (14.07)	14.56 (3.22)	*t*_28_=-0.611 a	0.546
Duration of alcohol use (years)	3.01 (1.63)	10.93 (3.71)	*U*=399.000	<0.001^*^
Alcohol use frequency (days per month)	5.80 (4.83)	9.78 (6.00)	*U*=283.500	0.023^*^
Alcohol units per typical drinking session^c^	9.81 (6.92)	8.43 (2.82)	*U*=190.000	0.799
Alcohol Use Disorders Identification Test	8.95 (5.53)	8.95 (4.82)	*U*=214.000	0.718

Abbreviations: NA, not applicable; THC, tetrahydrocannabinol.

a Levene's test for homogeneity of variance violated.

b Calculated only on those who had ever used cigarettes (*n=*34).

c Units used are standard UK units of alcohol; equivalent to 8 g of pure alcohol or ~3/5ths of a NIAAA standardized drink.

*P<0.05.

Values reflect mean (s.d.) unless otherwise stated; *P*-values reflect independent *t*-test comparing mean, Mann–Whitney *U*-test comparing median or chi-squared comparing frequency (as appropriate), by age group.
