# Supplementary material for: Are adolescents more vulnerable to the harmful effects of cannabis than adults? A placebo-controlled study in human males
Source: Transl Psychiatry. 2016 Nov 29;6(11):e961–. doi: 10.1038/tp.2016.225 (PMC5290352; doi:10.1038/tp.2016.225)
Supplement: Supplementary Tables [file tp2016225x1.docx]

Table S1. Frequency of positive/negative baseline session urine instant screen results, by age group.

|  | **Adolescents** | **Adults** |
| --- | --- | --- |
|  | n=18 | n=19 |
|  | pos/neg | pos/neg |
| **Amphetamine** | *0/18* | *0/19* |
| **Barbiturates** | *0/18* | *0/19* |
| **Benzodiazepines** | *0/18* | *1/18* |
| **Cocaine** | *0/18* | *3/16* |
| **MDMA** | *0/18* | *0/19* |
| **Methamphetamine** | *0/18* | *0/19* |
| **Methadone** | *0/18* | *0/19* |
| **Opiates** | *0/18* | *1/18* |
| **Oxycodone** | *0/18* | *0/19* |
| **Phencyclidine** | *0/18* | *1/18* |
| **THC** | *15/3* | *12/7* |

|  | **Adolescents** | | **Adults** | |
| --- | --- | --- | --- | --- |
|  | **Placebo** | **Cannabis** | **Placebo** | **Cannabis** |
|  | **Mean (SD)** | **Mean (SD)** | **Mean (SD)** | **Mean (SD)** |
| **Spatial N-back** | n=18 | | n=17 | |
| *Low load (1-back)* |  |  |  |  |
| Dicriminability | *3.28 (0.59)* | *2.75 (0.72)* | *3.28 (0.60)* | *2.94 (0.54)* |
| Reaction time correct trials (ms) | *536.74 (88.43)* | *565.36 (149.39)* | *532.16 (67.09)* | *633.55 (126.34)* |
| *High load (2-back)* |  |  |  |  |
| Dicriminability | *2.75 (0.91)* | *1.91 (0.93)* | *3.06 (0.63)* | *2.25 (1.14)* |
| Reaction time correct trials (ms) | *642.76 (135.29)* | *699.89 (170.10)* | *694.08 (174.09)* | *790.35 (222.01)* |
| **Prose recall** | n=20 | | n=20 | |
| Immediate | *6.80 (2.57)* | *4.70 (2.94)* | *6.53 (1.96)* | *4.03 (1.73)* |
| Delayed | *6.08 (2.68)* | *4.55 (2.89)* | *6.68 (1.90)* | *3.45 (1.81)* |
| **Stop-signal** | n=19 | | n=18 | |
| SSRT (ms) | *209.43 (64.95)* | *228.12 (67.35)* | *214.71 (46.51)* | *198.90 (43.61)* |
| Accuracy on no-signal trials | *0.990 (0.012)* | *0.965 (0.041)* | *0.989 (0.010)* | *0.986 (0.013)* |

Table S2. Means and standard deviations for spatial N-back, prose recall and stop-signal tasks, by drug and age group.
